# Generational challenges to radiology education and practice

**DOI:** 10.2349/biij.5.4.e31

**Published:** 2009-10-01

**Authors:** BJJ Abdullah

**Affiliations:** Department of Biomedical Imaging, University of Malaya, Kuala Lumpur, Malaysia

A number of sociologists and demographers have studied generational differences in-depth but despite this readily available information, individuals and organisations tend not to see their own lives as part of an era and are unaware of the characteristics of his/her generation [1].

Generational differences as shown in Table 1 [2] have real implications on how employers and employees, doctors and patients, teachers and students as well as journals and their readers interact. Each new generation brings a unique set of attitudes to the interactions which frequently do not fit the expectations of today’s leaders. For today’s organisations to fully benefit from the diversity of skills and perspectives of this upcoming generation, they must incorporate the Gen Y outlook into their cultures, organisation and management styles. It is also vital for the Gen Y to recognise and accept the other generations around them.

**Table 1:** Today’s workforce: Veterans or Pre-Boomers, Baby Boomers, Generation X and Generation Y [2].

	**Traditionalists/ Veterans 1925-1945**	**Baby Boomers 1946-1964**	**Generation X 1965-1980**	**Generation Y/ Millenials “Nexters” 1980 to 2002**
**Slogans**	“Keepers of the Grail”	Invented “Thank God, it’s Monday”	“Work to live, don’t live to work	“Upcoming optimists”
**Values**	Logic and Discipline	Participation / Equity	Balance between life and work	Diversity/Morals
**Provide**	Stable environment	Personal Challenges	Feedback	Structure
**Authority**	Respectful of authority	Nonauthoritarian	Dislikes Close Supervision	Respectful of Traditionalists
**Characteristics**	Conformers	Optimistic	Highly Motivated	Can-Do Attitude
**Work Priorities**	#1 Priority - Work	To Be a Star	Fun and Flexible	Money
**Train**	Don’t rush things	Skill practice	Visual Stimulation	Mentor Programs
**Technology**	Unsure and Resistant	Willing to Learn	Technologically Savvy	Technological Superior
**Career Goal**	Build a Legacy	Build a Stellar Career	Build a Portable Career	Build Parallel Careers

The majority of today’s college students are part of the Generation Y who considered to be the first human natives of the digital landscape [3]. These digital natives are characterised as:

- operating at twitch speed (not conventional speed)
- employing random access (not step-by-step)
- parallel processing (not linear processing)
- graphics first (not text)
- play-oriented (not work)
- connected (not stand alone)

They are also more ambitious and optimistic than Generation X, are the most ethnically diverse (35 percent are non-white), and favour different values and learning styles from their predecessors [4]. Additionally, Generation Y students face parental and self-pressure to study hard and excel, and they have proven to be up to the challenge. Today's students also expect to control "when, where, how, and how fast they learn." These students "perceive their learning environments as boundless," with most owning laptops that have sophisticated functions [5] and they no longer consider the physical library to be essential to their educational experience [6].

This generation is also responsible for a shift to user-driven innovation which has led to a democratisation of innovation processes, resulting in an explosive growth in products and features in various disciplines, particularly when combined with digital communications networks. There are groups of prosumers [7] who have created their own blogs, electronic journals, and discussion groups outside mainstream media, where they openly exchange their ideas and allow others to build on them. Many Gen Y doctors share their thoughts publicly via the so-called ‘Web 2.0' tools like blogs and social networking websites such as Twitter [8], Facebook [9], MySpace [10], and Bebo [11].

As Generation Y moves from their current position as medical students to become doctors, then onto specialists and leaders, they will bring with them these new challenging perspectives, collaborative and networking skills. Through their social online networks, these professionals not only discuss business ventures, successes and failures, but seek each other's advice in open mentoring opportunities and even share personal feelings in these virtual spaces.

Gen Y desire long-term relationships with employers, but on their own terms with work-life balance, better engagement with management, opportunities, responsibility and recognition of good work [2]. The medical profession is a different world from when Baby Boomers and even Generation X (in their 30s) graduated.

There is a profound gap in the perspectives and priorities that may exist between senior physicians and their young colleagues. Many older physicians and academics perceive young students and residents to be lazy, self-interested and pampered partly because they wish to have a clear separation between work and other parts of their life [12, 13]. For example these younger physicians prefer and expect fixed hours, a good call schedule with reliable coverage, and regular vacation time. The young doctors and students on the other hand, consider their mentors to be harsh, uncompromising and unaware of how the world has changed since they were medical students themselves. While the academic medical faculty acquires more than 70% of new information from printed journals, younger doctors are increasingly turning to the electronic page as can be seen with their iPhones, Blackberries, 3G mobile phones, and netbooks. The end result is a festering hostility in hospitals, medical practices and training programs.

Such fundamental differences must be recognised by the educators, leaders, managers and editors in order to ensure they stay relevant or else they face becoming the most recent addition to the heaps of dinosaurs from all the eras before us. Parents and adults are no longer the competent leaders of the changing society, but rather the uncomfortable facilitators trying to keep abreast with the rapidly changing technology and other social, economic, political and environmental issues, including the all-encompassing effects of globalisation.
